# Can social protection contribute to social connectedness in contexts of forced displacement and crisis? Lessons from Jordan’s labelled cash transfer for education

**DOI:** 10.1016/j.worlddev.2024.106886

**Published:** 2025-04

**Authors:** Bassam Abu Hamad, Nicola Jones, Shoroq Abuhamad, Sarah Baird, Erin Oakley

**Affiliations:** a Al-Quds University, School of Public Health, 101, Tel El Hawa Gaza, Palestine; b ODI and Gender and Adolescence: Global Evidence (GAGE), UK; c Gender and Adolescence: Global Evidence (GAGE), Palestine; d The George Washington University Milken Institute School of Public Health, USA

**Keywords:** Social protection, Cash transfers, Refugees, Adolescence, Social cohesion, Connectedness

## Abstract

Social protection programming can help address gender- and lifecycle-specific vulnerabilities, particularly in humanitarian contexts. Although adolescents are disproportionately affected by crises, there remains limited evidence about how such programming can mitigate risks they face, including social isolation. This article explores how a ‘cash-plus’ social protection response enhances refugee adolescents’ social connectedness and contributes to broader social cohesion and sustainable peace.

Mixed-methods data collected during the Covid-19 pandemic with 996 Syrian adolescents living in Jordanian host communities involved beneficiaries and non-beneficiaries of *Hajati,* UNICEF’s unconditional cash transfer. Explicitly labelled to promote adolescent education, Hajati also encourages enrolment in an adolescent empowerment programme, *Makani* (My Space), designed to foster resilience and social connectedness.

Survey results were mixed, finding evidence of modest improvements among beneficiaries on a subset of social connectedness and resilience outcomes. *Hajati* beneficiaries (particularly girls and older adolescents, aged 15–18) report higher levels of family support and better coping with pandemic stressors. Among younger adolescents (12–14) and girls, beneficiaries were more likely to report having a trusted adult, and greater social support from non-family adults. Beneficiary status was not associated with measures of peer connectedness or perceived social cohesion. Qualitative interview findings, however, indicate beneficiaries enjoy stronger peer networks, partly due to participating in group-based programming. Overall, programme participation helped girls (and, less so, boys) cope with pandemic stressors, while strengthening adolescent social connectedness through opportunities for in-person and online interaction.

We conclude with suggestions on how to strengthen programming to promote social cohesion and sustainable peace.

## Highlights

• Beneficiaries of *Hajati* report higher levels of family support during the pandemic.
• Beneficiaries of *Hajati* report better coping with pandemic stressors.
• *Hajati* beneficiaries report having greater social support from non-family adults.
• Beneficiaries of *Hajati* program enjoy stronger peer networks.

## 1 Introduction

An emerging body of evidence suggests that social protection programming (including cash transfers) can be a critical component in promoting the well-being of populations in humanitarian and post-conflict settings (Jeong & Trako, 2022). Cash transfers, in particular, not only help households meet basic survival needs but they can also help reconnect displaced adolescents with the formal education system (Aurino & Giunti, 2022) and improve individuals’ mental health and subjective well-being (McGuire et al., 2022, Quattrochi et al., 2020). The effects of cash and in-kind social protection programmes on community social cohesion, social connectedness (i.e. horizontal relationships with and a sense of belonging to family, peers and community) (van Bel et al., 2009), and social inclusion of refugee households are less well understood – partly because few studies examine these types of outcomes (Jeong & Trako, 2022).

The limited evidence that does exist finds some positive impacts of cash and in-kind transfers on social cohesion (Jeong and Trako, 2022, Valli et al., 2019). Other studies have conceptualised social cohesion using a variety of measures, such as: group membership and social participation; attitudes of acceptance of diversity; recipient-reported trust in other community members; giving and receiving of support to and from other households; self-reported contributions to the community; and negative experiences that recipients may have had such as theft, discrimination or experiencing ‘problems’ with others in the neighbourhood (Quattrochi et al., 2020, Valli et al., 2019, Lehmann and Masterson, 2014).

Although not focusing specifically on social protection programming, Strang and Quinn (2021) introduce a novel framework to conceptualise social connectedness in the context of refugee integration that goes beyond binary distinction between bridging (vertical connections – e.g. between citizens and local leaders) and bonding (horizontal connections – e.g. among people from different ethnic or nationality backgrounds) social capital. They look at three different domains of social connections: social bonds (trusted relationships, primarily with family members and groups with a shared ethnicity or nationality); social bridges (with people who are different and outside these immediate social circles); and social links (with state institutions). However, we found little focus in the literature on the impact of social protection on adolescent-specific experiences of social cohesion and social connectedness, despite the fact that adolescence is a critical stage for emotional and social-emotional development (Ross et al., 2020). Indeed, a growing body of research underscores that family, peer and community connectedness are particularly important during adolescence due to neurodevelopment and social maturation processes (Blum et al., 2022).

The research on which this article is based is part of the Gender and Adolescence: Global Evidence (GAGE) 10-year longitudinal study exploring the gendered experiences of adolescents aged 10–19 years. In this article, we examine the social inclusion and cohesion outcomes associated with a UNICEF social protection programme, *Hajati*, which targets households with children and adolescents enrolled in school under 18 years of age and living in host communities in Jordan. The young people participating in *Hajati* were also given information about and encouraged to enrol in services provided by UNICEF Jordan’s *Makani* (‘My Space’) programme, which operates through one-stop community centres (see Banati et al., 2021 for further details). We explore the impact of the *Hajati* cash transfer programme on Syrian refugee adolescents’ experiences of resilience, social connectedness, and community social cohesion during the pandemic.

The article begins by situating our findings in the broader literature, underscoring the dearth of evidence from settings affected by forced displacement. We then describe our methodology. The results section discusses our quantitative and qualitative findings on how social protection can support social cohesion, connectedness and resilience among adolescents. The article concludes by discussing the implications of our findings for policy and programming, including in the context of the Sustainable Development Agenda and Sustainable Development Goal (SDG) 16, which commits to promoting just, inclusive and peaceful societies.

## 2 Theoretical framing

Social protection programming is increasingly seen as a way to transform social relations (Schüring & Loewe, 2021). There is widespread agreement that it can have a transformative impact by addressing the root causes of poverty, such as gender inequality and social exclusion (Burchi et al., 2022, Molyneux et al., 2016, Holmes and Jones, 2013, Sabates-Wheeler and Devereux, 2007). These goals are reflected in UNICEF’s (2019) distilled definition of social protection as “a set of policies and programmes aimed at preventing or protecting all people against poverty, vulnerability and social exclusion throughout their life-course, with a particular emphasis towards vulnerable groups”.

The cash or asset transfers and public works programmes that form the backbone of social protection in low- and middle-income country (LMIC) contexts have been shown to be positively associated with an array of outcomes, including reducing poverty, improving access to employment, and reducing violence (Jones et al., 2021;. Save the Children, 2018). Yet because the effects of social protection programmes are often more limited than intended – in terms of scale and durability as well as social transformation – the past decade has seen growing interest in a category of initiatives known as ‘cash-plus’ programming (Roelen et al., 2017), which recognise that addressing consumption poverty is often not sufficient to improve broader well-being. Cash-plus programming provides cash alongside complementary programming such as psychosocial support, behaviour change communication, or linkages to existing services (ibid.). Although there is still limited evaluation evidence of cash-plus programming, nascent evidence suggests that it can have positive impacts, especially on children’s well-being (Little et al., 2021).

As part of broader interest in the transformative potential of social protection, there has been growing academic and policy attention to how programming can be leveraged to improve social connectedness and social cohesion (Burchi et al., 2022, Koehler, 2021, Loewe et al., 2019). Defined by Leininger et al. (2021: 3) as “a set of attitudes and norms that includes trust, an inclusive identity and cooperation for the common good”, and informally characterised as “the glue that holds society together”, social cohesion is seen as a key development objective because it can improve resilience to shocks, reduce violence, and contribute to broader community development (Burchi et al., 2022). In some contexts, social protection has been found to strengthen the horizontal relationships between people, primarily by fostering dignity and participation and improving trust and inclusion; in other contexts, it has strengthened the vertical relationships between people and the state (Burchi et al., 2022, Mahmud and Sharpe, 2021, Molyneux et al., 2016, Babajanian, 2012).

Historically, development and humanitarian contexts have seen different approaches to social protection (Dafuleya et al., 2022, Cherrier, 2021, Ghorpade and Ammar, 2021). In LMICs, as noted earlier, programmes are designed around poverty; they aim to address shocks resulting from lifecycle events (such as illness or unemployment), tend to be relatively low value, and are primarily delivered by governments (ibid.). In humanitarian contexts, programmes are designed to save lives and alleviate suffering; they aim to address short-term external shocks (such as conflict or drought), tend to be higher value, and are primarily delivered by donors and non-governmental organisations (NGOs) (ibid.).

However, as natural disasters become more frequent due to climate change, and as displacement becomes more common and more protracted, development and humanitarian approaches to social protection are beginning to converge (UNHCR, 2023, Organisation for Economic Co-operation and Development (OECD)/ Expert Group for Aid Studies of the Government of Sweden (EBA), 2022, Jeong and Trako, 2022, Cherrier, 2021; European Commission, 2019).

Indeed, recognising the need to protect the gains made in high-income countries and to strengthen and expand the social protection offered in fragile contexts, the 2016 World Humanitarian Summit produced an agreement to deliver aid in a manner that supports both refugees and host communities – working through local and national service providers wherever possible, rather than running parallel systems (European Commission, 2019, Inter-Agency Standing Committee (IASC), 2016).

The adoption of development approaches in humanitarian contexts is evident on many fronts. For example, cash transfers have largely replaced direct food support, and humanitarian actors – while continuing to provide informal learning opportunities for children who cannot access school – are now funding transfers (some accompanied by complementary components) aimed at supporting children and adolescents’ access to formal education (Dafuleya et al., 2022, UNHCR, 2022, UNHCR, n.d.). While the evidence is thin, and even more mixed than that generated in development contexts, it suggests that development approaches to social protection in humanitarian contexts can improve food security and nutrition but also well-being and access to education (Jeong & Trako, 2022; Aurino & Giunti, 2021; Doocy and Tappis, 2018, Gentilini, 2016). In Lebanon, for example, a cash transfer labelled for education has improved Syrian children’s enrolment and food consumption, and has positively impacted their aspirations for the future and their psychosocial well-being (de Hoop et al., 2019).

Unsurprisingly, there is substantial interest in using social protection to improve social connectedness and social cohesion and to promote peace in crisis-affected contexts, where competition for scarce resources (such as housing and jobs) can be accompanied by resentment when refugees are felt to be receiving more assistance than host populations, and where the necessity of integration is increasingly recognised given low rates of return and resettlement (Dafuleya et al., 2022, Lowe et al., 2022, Cherrier, 2021, Carpenter et al., 2012). Research in this area is informed by notions of ‘positive peace’, which underscore that peace is not just about the absence of violence (negative peace) but also about constructing the attitudes, institutions and structures that create and sustain positive peace. This includes the absence of structural violence (political and economic conditions that lead to the exploitation and repression of certain groups of people in a society) and cultural violence (that normatively justifies either physical or structural violence) (Galtung & Fischer, 2013), and is instead characterised by social justice, fairness and egalitarianism (Galtung, 1996). As a result, some humanitarian interventions are designed to match existent government interventions provided to national citizens while others are designed to equitably serve vulnerable households, regardless of their citizenship status. For example, the educational cash transfer for refugees living in Turkey deliberately mirrors the transfer provided by the government to Turkish children, in terms of its benefits and conditions (Ring et al., 2020). In Lebanon, food vouchers initially available only to Syrian families are now also provided to poor Lebanese families (Idris, 2019).

Yet evidence of impact is rare, largely because social cohesion and social connectedness are difficult to measure. There is, however, some evidence to suggest that social protection interventions can have positive influences on both horizontal and vertical social connectedness and cohesion in humanitarian contexts (Jeong and Trako, 2022, Lowe et al., 2022). In Ecuador, evidence from a cluster randomised study found that a cash transfer provided to Colombian refugees and poor Ecuadorians improved social cohesion in treatment communities, especially in terms of individuals’ relationships with one another (Valli et al., 2019). In a qualitative study of a similar programme in Jordan, where a cash-for-work intervention targeted both Syrian refugees and vulnerable Jordanians, findings suggested that it strengthened cooperation and relationships between the two groups (Zintl & Loewe, 2022). Lowe et al. (2022) emphasise that to maximise impacts on cohesion, it is vital that actors raise community awareness about how social protection benefits are distributed.

## 3 Context in Jordan

Jordan’s population has increased rapidly over the past decade, reaching 11.3 million in 2022, a third of which are refugees (Department of Statistics, 2022). There are more than 2 million registered Palestinian refugees (most of whom have citizenship) who have been in Jordan for decades (ibid.). Of the 1.3 million Syrians in Jordan, half are registered refugees with UNHCR, and most have been in Jordan for a decade. Nearly a quarter of Jordan’s population are adolescents (Macrotrends, 2023).

Compared to other countries in the region, integration efforts have been largely positive, and as Zintl and Loewe (2022) note, social cohesion has been facilitated by the fact that many Syrian refugees are from the same tribe as those living in the north of Jordan. Even so, significant inequalities remain. Unemployment in the country is exceptionally high, especially for refugees, and poverty rates have climbed among citizens and refugees alike over the past decade (ibid.). Moreover, despite scaling up free education, primary education is not yet universal, with Syrian children particularly likely to be out of school (Abu Hamad et al., 2021).

The Jordanian government, backed by the international community, has made substantial efforts to support refugees. The main social protection cash programmes are: the National Aid Fund, which targets poor Jordanian families (approximately 92,000); UNHCR social assistance, which targets poor Syrian refugees in host communities (32,000 families); and the *Hajati* cash-for-education labelled transfer which targets households with children and adolescents under 18 years attending school from refugees as well as vulnerable host communities (UNICEF, 2021, Abu Hamad et al., 2017). Syrians also receive support from the World Food Programme (WFP), while those living in formal refugee camps have free shelter, access to education and basic health care. The United Nations Relief and Works Agency for Palestine Refugees in the Near East (UNRWA) provides education and health services (but little social protection) for Palestinian refugees. UNICEF’s *Makani* (My Space) programme provides non-formal education support, life skills and protection services to approximately 100,000 children and adolescents of all nationalities, and referrals back to school for *Hajati* beneficiaries (UNICEF, 2021′ Presler-Marshall et al., 2022).

Not surprisingly, the Covid-19 pandemic, which resulted in a protracted lockdown and the closure of schools for approximately 18 months (between March 2020 and August 2021), exacerbated the challenges facing Jordan. The pandemic was not only a health and economic crisis, but also placed a strain on social bonds within communities, and especially between refugee and host communities (Jones et al., 2022).

### 3.1 *Hajati* labelled cash transfer programme

The *Hajati* programme, established by UNICEF in 2017, provides beneficiary households with a monthly cash amount (20–25 Jordanian dinar (JD), equivalent to US$28–36) per eligible school-aged child, for up to 4–6 children (UNICEF, 2021). It was designed as a ‘labelled cash transfer’; although unconditional (households did not need to prove school enrolment or attendance), UNICEF provided clear messaging that the cash was intended to support children’s enrolment and retention in education, and to mitigate negative coping mechanisms such as child labour and child marriage (UNICEF Office of Research, 2021). The programme targeted displaced Syrian refugees living in host communities and informal tented settlements, Palestinian refugees living in host communities and refugee camps, and vulnerable Jordanian households. The programme used a child-sensitive multi-dimensional vulnerability targeting approach involving 16 indicators spanning education, health, shelter, water and sanitation (UNICEF, 2021). In 2019–20, *Hajati* supported approximately 11,000 children and adolescents (ibid.). When schools closed in March 2020 due to the onset of the Covid-19 pandemic, UNICEF expanded the programme to support more than 30,000 children and adolescents (from birth up to 18 years) (UNICEF Office of Research, 2021). *Hajati* has established close links to *Makani,* whereby *Hajati* beneficiaries were actively encouraged to participate in *Makani* programming, and *Makani* facilitators also supported *Hajati* beneficiaries to re-enter formal schooling (Abu Hamad et al., 2021).

## 4 Materials and methods

This article draws on findings from a mixed-method research study undertaken in Jordan as part of the GAGE multi-country longitudinal research programme. The study includes qualitative and quantitative components.

### 4.1 Quantitative data

At baseline in 2018, GAGE collected data on a cohort of 4,101 Jordanian, Syrian and Palestinian adolescent boys and girls living in Jordanian host communities, refugee camps and informal tented settlements. At baseline, adolescents were randomly sampled from databases of vulnerable adolescents and families compiled and maintained by UNICEF and UNHCR across five governorates in northern Jordan, with deliberate oversampling and additional purposive sampling for particularly vulnerable adolescents – those who married as children and those with disabilities (for additional details on this sample see Jones et al., 2019). This article focuses on respondents to the GAGE Covid-19 Round 2 (R2) phone-based survey. Implemented by trained enumerators from the Amman-based survey research organisation, Mindset, the survey was carried out between October 2020 and January 2021.

For this article, we focus on respondents who are Syrian refugee adolescents living in host communities, excluding those Syrian refugees living in camps who are not eligible for *Hajati* cash assistance, and those in informal tented settlements, due to a small sample size. To ensure that we examine only individuals who are eligible to receive *Hajati*, we further restrict the sample to those adolescents aged 12–18 years at the time of the phone survey. Applying this criterion to the baseline data (after calculating the expected age of adolescents at the endpoint of the phone survey), 1,319 Syrian adolescents interviewed during the baseline survey were eligible to be included in the current analysis; excluded participants were either camp residents (n = 929), those living in informal tented settlements (n = 271), not of Syrian nationality (n = 1,257), or were over the age of 18 and no longer eligible for *Hajati* (n = 325).Of the 1,319 eligible adolescents, we obtained complete data from 996 adolescent–caregiver dyads (a 75.5 % response rate compared to baseline). Comparing adolescent and household characteristics between the baseline sample and those followed up during the Covid-19 phone survey, we found the samples were similar based on adolescent gender, disability status, marital status, household assets, head-of-household gender, and baseline school enrolment (see Table S1); however, the follow-up rate was slightly lower among adolescents who were in the younger cohort at baseline (age 10–14).

### 4.2 Qualitative data

The qualitative data sought to explore, in greater depth, how social protection programmes have contributed to reducing vulnerabilities (including social isolation and exclusion), and promoting resilience and social connectedness. The data was triangulated to produce a multi-layered analysis, enabling us to capture adolescents’ experiences and insights into how to maximise the contribution of social protection programmes. Qualitative data collection complemented the findings from the Covid-19 phone survey. Individual interviews with adolescents allowed researchers to assess how the pandemic had impacted participants, according to their own perceptions. We interviewed 52 adolescents aged 12–18 years, including 4 married girls and 5 adolescents with disabilities, and conducted 4 focus group discussions with parents in host communities involving a total of 24 parents. We also interviewed 22 key informant experts from the social services sector.

### 4.3 Research instruments

The Covid-19 phone survey was designed to assess adolescent vulnerabilities and experiences, and how these had changed during the pandemic. It included questions on household income, access to social protection programming, school enrolment, food security levels, physical and mental well-being, and sources of family and social support (Baird et al., 2020). Moreover, to measure coping and resilience levels, we used the Brief Resilient Coping Scale (BRCS), a 4-item scale (0 to 16) that measures tendencies to cope with stress in a highly adaptive manner (Sinclair & Wallston, 2004) (see Table 1).

**Table 1:** Outcomes across three domains.

**Outcome**	**Type of outcome**	**Description of measure**
**Domain 1: Adolescent resilience**		
The adolescent reports coping well with pandemic-related stress	Binary	The adolescent confirms that the statement “I am coping well with the difficulty and stress caused by the Covid-19 pandemic” describes themselves (well or very well).
Brief Resilient Coping Scale	Continuous	A 4-item scale (0 to 16) that measures tendencies to cope with stress in a highly adaptive manner (Sinclair & Wallston, 2004); higher scores indicate a higher level of resilient coping.
Low resilient coping score	Binary	A binary measure of low resilient coping drawn from the 4-item BRCS, a score of between 0 and 9 indicates low resilient coping.
High resilient coping score	Binary	A binary measure of high resilient coping drawn from the 4-item BRCS, a score of between 13 and 16 indicates high resilient coping.
**Domain 2: Social connectedness**		
Connection to peers	Binary	The adolescent confirms that they have at least one trusted friend outside of the household.
Connection to peers during the pandemic	Binary	The adolescent confirms that the statement “My friends offer me help and assistance to overcome the difficulty and stress caused by Corona/ Covid-19 pandemic” describes themselves (well or very well).
Connection to trusted adults	Binary	The adolescent confirms that they have at least one trusted adult (either within or outside of their household).
Connection to trusted adults during the pandemic	Binary	The adolescent confirms that the statement “Adults outside my family (such as community members, sports coaches, religious leaders, or teachers) are helping me cope with the difficulty and stress caused by the Corona/Covid-19 pandemic” describes themselves (well or very well).
Support from family during the pandemic	Binary	The adolescent confirms that the statement “My family is helping me cope with the difficulty and stress caused by the Corona/Covid-19 pandemic” describes themselves (well or very well).
**Domain 3: Perceived social cohesion**		
Adolescent perception of social cohesion in their community during the pandemic	Binary	The adolescent agrees or partially agrees with the statement: “People in my community, regardless of nationality, ethnicity, and religion, are coming together to help each other during the current health crisis” (adolescents aged 15–18 only).

We used dynamic qualitative tools, including vignettes and diaries, in addition to semi-structured in-depth interviews (IDIs) and key informant interviews (KIIs) to explore adolescents’ lived experiences, their coping mechanisms, experiences of violence, intra-household relations, digital connectivity and online behaviours, community relationships (such as volunteerism), and views on authorities’ responses to the Covid-19 pandemic.

### 4.4 Data analysis

Cleaning and analysis of the quantitative data was completed in Stata 16.1. We begin our analysis by providing descriptive statistics (means/proportions and counts) for the demographic characteristics of adolescents included in our sample. Then, we present descriptive statistics for outcomes of interest, disaggregated by *Hajati* beneficiaries and non-beneficiaries, and by gender and age cohort, as well as for the two subgroups of interest (adolescents with disabilities and girls who married as children). For the current analysis, we consider 10 outcomes across 3 domains of interest at the intersection between social protection and social cohesion: adolescent resilience; social connectedness; and perceived social cohesion. Specific outcomes are outlined in Table 1.

After presenting summary statistics, we begin our formal analysis with multivariate regression to estimate the difference in outcomes associated with household receipt of *Hajati* cash support. For binary outcomes, we employ a multivariate linear probability model (for ease of interpretation). For our continuous outcome (BRCS score), we use a multivariate linear regression. All multivariate analyses are adjusted for baseline household assets,^1^ based on an asset index (a locally adapted list of assets owned by the household modelled after the methods of Filmer & Pritchett, 2001) converted into deciles within the sample (1–10). We also adjust for two other baseline household characteristics that may influence outcomes: household size; and whether the household is female-headed. Finally, we adjust for a set of adolescent characteristics collected during the phone survey: adolescent gender; adolescent age cohort (12–14 years and 15–18 years); adolescent marital status; and adolescent disability status. We repeat this multivariate analysis disaggregated by gender (male and female) and age cohort (younger adolescents and older adolescents) to identify any associations specific to these subgroups.

Finally, to ensure the most appropriate comparison between *Hajati* beneficiaries and non-beneficiaries, we conducted an additional, complementary analysis using nearest-neighbour matching. Because *Hajati* beneficiaries are selected based on an index of vulnerability measures (UNICEF Office of Research, 2021), these adolescents tend to be from the most vulnerable households. Therefore, we employ a matching strategy to compare beneficiary adolescents to non-beneficiary adolescents with the most similar demographic and household characteristics. A matching strategy can be a useful way to capture average treatment effect on the treated in cases where a set of covariates that may predict outcomes are distributed differently in the treatment and comparison groups (Stuart, 2010). We use nearest-neighbour matching to identify two matches for each *Hajati* beneficiary using a set of baseline household characteristics (including asset decile drawn from the asset index described earlier, household size, and whether the household is female-headed), and current adolescent characteristics (including gender, age cohort (12–14 or 15–18 years), disability status, and marital status). We required an exact match for age cohort and gender. We repeated our matching analysis by subgroup based on gender and age cohort to examine outcomes associated with household receipt of *Hajati* transfers within each subgroup.

Specifically, we used the ‘teffects’ command in Stata, which computes robust Abadie-Imbens standard errors (Abadie & Imbens, 2016). For each matching analysis, we assessed the performance of the matching strategy; the literature suggests that matched standardised differences should be less than 0.25 (Stuart, 2010) or, more ideally, less than 0.10 (Hui et al., 2023), and variance ratios for each covariate should be between 0.5 and 2.0 (and ideally close to 1.0) when matching is used (Stuart, 2010). All respondents find matches within the specified distance (using Mahalanobis distance for non-exact matches), and the matched standardised differences are minimal (largest is 0.062), while the variance ratio is generally close to 1 (ranges from 0.806 to 1.076) for outcomes that include the full sample. Matching is less viable when examining smaller subgroups. For example, the matched standardised differences tend to be larger and variance ratio takes a wider range in comparisons made among the older cohort only (largest standardised difference = 0.056, variance ratio range = 0.757 to 1.048), among the younger cohort only (largest standardised difference = 0.105, variance ratio range = 0.789 to 1.092), among girls only (largest standardised difference = 0.051, variance ratio range = 0.522 to 1.064), and among boys only (largest standardised difference = 0.103, variance ratio range = 0.803 to 1.14), likely due to the smaller sample sizes. The comparison for these subgroups should, therefore, be interpreted with caution. We do not present the results of the matching analysis by both cohort and gender at the same time (i.e., for the outcome that is restricted to the older cohort) due to the smaller sample size and inadequate performance on balance checks.

The qualitative interviews were recorded, transcribed, translated and then thematically coded using a coding book informed by the GAGE conceptual framework (GAGE consortium, 2019) and focusing on different dimensions of adolescent social connectedness, including family, peer, community and online networks, as well as a sense of belonging to each of these domains. Coding was completed using MAXQDA 12 software.

### 4.5 Ethics

The research followed the international code of ethics (World Health Organization, 2017) and permissions for the study were sought and granted from the relevant bodies in Jordan. Research ethics approvals were also obtained from the George Washington University Committee on Human Research Institutional Review Board (071721). Approval was also granted by Jordan’s Ministry of Interior and the Department of Statistics. We also secured an Institutional Review Board approval from the Overseas Development Institute’s ethics review (02438) and from the World Health Organization Research Ethics Review Committee.

Verbal consent was obtained from participants aged 18 years and over and emancipated minors, and verbal assent was sought for those under 18 years, as well as verbal consent from their caregiver (typically the primary female caregiver). Enumerators were oriented on how to interact with adolescents in an age-appropriate and gender-sensitive way.

### 4.6 Limitations of the study

It is important to point out some limitations of our data and analysis. Self-reported measures may contribute to recall bias as participants are sometimes unable to describe their experiences, feelings and attitudes accurately. Thus, the responses are indicative of perceived effects/impacts rather than causal relationships. Another limitation is that phone-based interviews are, by necessity, shorter than face-to-face interviews, which limits the depth of information that can be elicited. Participants (particularly adolescent girls) may also have had limited privacy during the phone calls with other family members present, although researchers had received training to address such challenges, including briefing caregivers prior to the interview the importance of interviewee privacy and emphasising to the adolescents that they could reschedule the interview to a time when they would likely have greater privacy. In addition, while it might be the case that in a semi-authoritarian state like Jordan that respondents could be concerned about phone line tapping – and perhaps especially refugees who had experienced a civil war in Syria –the interview questions did not contain politically sensitive content and thus this risk seems minimal. Moreover, the use of mixed-methods data and using more than one dataset might have helped to overcome these limitations. It is also the case that any potential data quality risks associated with phone interviews would not have been different between beneficiaries and non-beneficiaries.

## 5 Results

We begin by presenting quantitative evidence on the 996 adolescent–caregiver dyads from Syrian refugee households, including 143 *Hajati* beneficiaries and 853 non-beneficiaries, regarding adolescent individual resilience and social connectedness. We then discuss the qualitative evidence and the insights it provides into underlying change pathways.

Overall, adolescents from beneficiary and non-beneficiary households had similar characteristics (Table 2). Approximately half of *Hajati* beneficiaries and non-beneficiaries were female, and approximately 15 %–16 % of each group were adolescents with disabilities. Among adolescent girls, approximately 10 % of beneficiaries and non-beneficiaries were married. *Hajati* beneficiaries were slightly younger than non-beneficiaries, with 62 % of beneficiaries in the younger cohort (aged 12–14 years) compared to 60 % of non-beneficiaries; however, the mean age of each group at the time of the survey was almost equivalent, at 14.78 years (sd 2.02) and 14.83 years (sd 2.21), respectively. Notably, *Hajati* beneficiaries were only slightly more likely to be enrolled in school in March 2020 prior to school closures (84 % compared to 81 %).

**Table 2:** Sample characteristics – Syrian refugees living in host communities.

**Characteristics**±	***Hajati* beneficiaries (n = 143)**		**Non-beneficiaries (n = 853)**	
	**Count**	**%**	**Count**	**%**
Adolescent characteristics				
=1 if adolescent is female	72	0.503	419	0.491
=1 if adolescent is male	71	0.497	434	0.509
=1 if adolescent is in younger cohort (age 12–14)	89	0.622	512	0.600
=1 if adolescent is in older cohort (age 15–18)	54	0.378	341	0.400
=1 if adolescent has a functional disability	21	0.147	134	0.158
=1 if adolescent has no disability	122	0.853	715	0.842
=1 if adolescent was ever married (girls only)	7	0.099	43	0.103
=1 if adolescent was never married (girls only)	64	0.901	376	0.897
=1 if adolescent enrolled in formal school in March 2020 (before school closures)	120	0.839	690	0.809
=1 if adolescent not enrolled in formal school in March 2020	23	0.161	163	0.191
=1 if adolescent was *Makani* participant at most recent round available	28	0.196	160	0.188
Household characteristics				
=1 if household had above-median assets at baseline †	43	0.301	291	0.341
Baseline asset decile (mean) †	−-	4.098	−-	4.436
Baseline household size (mean) †	−-	7.775	−-	7.105
Female-headed household †	50	0.352	175	0.207
=1 if household receives UNHCR cash benefits	70	0.507	297	0.354
=1 if household receives a WFP food voucher card	134	0.971	812	0.967

Notes: This table presents counts and percentages of respondents based on household characteristics. Outcomes marked with a symbol “†“ are drawn from baseline data collection (conducted in late 2018 to early 2019). The remaining outcomes are drawn from the Covid R2 phone survey, conducted between October 2020 and January 2021.

± Note that the counts and percentages presented here exclude any adolescents or household respondents who responded “don’t know” or “refused” for the relevant question.

Household characteristics reveal several key differences between beneficiaries and non-beneficiaries. First, beneficiary households appear to have slightly lower baseline assets than non-beneficiary households, suggesting a higher level of poverty at baseline. Second, beneficiary households on average tend to be slightly larger than non-beneficiary households (mean of 8 members compared to 7). Beneficiary households were more likely to have a female head of household (35 % compared to 21 % among non-beneficiaries) and to receive additional cash aid (not targeted to adolescents or school enrolment) from the UNHCR cash aid programme for Syrian refugees (51 % compared to 35 %). Both groups observe high coverage of WFP food aid voucher cards (97 % across the sample).

We next present the overall means for social connectedness and social cohesion outcomes across beneficiary and non-beneficiary adolescents (Table 3). Just over half of adolescents reported that they had a trusted friend in their lives (56 %), while 65 % reported having at least one trusted adult. The vast majority of adolescents reported that their family was helping them to cope with the stresses of the pandemic (84 %), with slightly higher unadjusted rates among *Hajati* beneficiaries (90 %) than non-beneficiaries (84 %). Fewer adolescents reported feeling supported in dealing with pandemic stress by friends (62 %) or other adults outside of the household (43 %). Even so, 66 % of adolescents reported coping well with pandemic stress overall. Based on our measure of resilience (BRCS), approximately 34 % of adolescents scored within range for low resilient coping, while 15 % scored within range for high resilient coping.

**Table 3:** Summary statistics: outcomes for *Hajati* beneficiaries and non-beneficiaries and by subgroup.

**Outcome**±	All adolescents			Younger adolescents (age 12–14)		Older adolescents (age 15–18)		Adolescent girls		Adolescent boys		Adolescents with disabilities*		Married adolescent girls*	
	Overall	*Hajati Ben.*	Non-*Ben*	*Hajati Ben.*	Non-*Ben*	*Hajati Ben.*	Non-*Ben*	*Hajati Ben.*	Non-*Ben*	*Hajati Ben.*	Non-*Ben*	*Hajati Ben.*	Non-*Ben*	*Hajati Ben.*	Non-*Ben*
	mean(sd)	mean(sd)	mean(sd)	mean(sd)	mean(sd)	mean(sd)	mean(sd)	mean(sd)	mean(sd)	mean(sd)	mean(sd)	mean(sd)	mean(sd)	mean(sd)	mean(sd)
=1 if adolescent reports they have a friend that they can trust	0.559 (0.497)	0.552 (0.499)	0.560 (0.497)	0.517 (0.503)	0.502 (0.500)	0.611 (0.492)	0.648 (0.478)	0.542 (0.502)	0.547 (0.498)	0.563 (0.499)	0.574 (0.495)	0.714 (0.463)	0.485 (0.502)	0.429 (0.535)	0.465 (0.505)
=1 if adolescent reports they have an adult that they can trust	0.651 (0.477)	0.650 (0.479)	0.651 (0.477)	0.697 (0.462)	0.598 (0.491)	0.574 (0.499)	0.730 (0.445)	0.708 (0.458)	0.642 (0.480)	0.592 (0.495)	0.659 (0.475)	0.762 (0.436)	0.649 (0.479)	0.571 (0.535)	0.721 (0.454)
=1 if adolescent's friends are helping them cope with the pandemic	0.619 (0.486)	0.664 (0.474)	0.612 (0.488)	0.663 (0.475)	0.604 (0.49)	0.667 (0.476)	0.625 (0.485)	0.708 (0.458)	0.604 (0.490)	0.620 (0.489)	0.620 (0.486)	0.524 (0.512)	0.567 (0.497)	0.857 (0.378)	0.581 (0.499)
=1 if other adults outside the family are helping them cope with the pandemic	0.426 (0.495)	0.469 (0.501)	0.419 (0.494)	0.449 (0.500)	0.421 (0.494)	0.500 (0.505)	0.416 (0.494)	0.528 (0.503)	0.376 (0.485)	0.408 (0.495)	0.461 (0.499)	0.333 (0.483)	0.388 (0.489)	0.571 (0.535)	0.442 (0.502)
=1 if family is helping them to cope with pandemic stress	0.844 (0.363)	0.902 (0.298)	0.835 (0.372)	0.876 (0.331)	0.838 (0.369)	0.944 (0.231)	0.829 (0.377)	0.944 (0.231)	0.811 (0.392)	0.859 (0.350)	0.857 (0.351)	0.857 (0.359)	0.821 (0.385)	1.00 (0)	0.860 (0.351)
=1 if adolescent is coping well with pandemic stress	0.656 (0.475)	0.762 (0.427)	0.638 (0.481)	0.764 (0.427)	0.626 (0.484)	0.759 (0.432)	0.657 (0.475)	0.806 (0.399)	0.647 (0.479)	0.718 (0.453)	0.630 (0.483)	0.810 (0.402)	0.541 (0.500)	0.714 (0.488)	0.698 (0.465)
BRCS total score (0–16)	10.148 (2.987)	10.559 (2.957)	10.079 (2.988)	10.270 (2.791)	9.665 (3.075)	11.037 (3.180)	10.698 (2.743)	10.778 (3.003)	9.813 (3.012)	10.338 (2.913)	10.334 (2.946)	11.095 (2.982)	9.776 (3.267)	10.857 (4.981)	10.628 (3.170)
=1 if adolescent scored within low resilient coping range (0–9), BRCS	0.342 (0.475)	0.280 (0.450)	0.352 (0.478)	0.337 (0.475)	0.411 (0.492)	0.185 (0.392)	0.264 (0.441)	0.292 (0.458)	0.361 (0.481)	0.268 (0.446)	0.343 (0.475)	0.286 (0.463)	0.403 (0.492)	0.143 (0.378)	0.256 (0.441)
=1 if adolescent scored within high resilient coping range (13–16), BRCS	0.154 (0.361)	0.161 (0.369)	0.153 (0.36)	0.157 (0.366)	0.137 (0.344)	0.167 (0.376)	0.176 (0.381)	0.208 (0.409)	0.132 (0.338)	0.113 (0.318)	0.173 (0.379)	0.333 (0.483)	0.149 (0.358)	0.286 (0.488)	0.233 (0.427)
=1 if adolescent agrees or partially agrees with the statement: “People in my community, regardless of nationality, ethnicity, and religion, are coming together to help each other during the current health crisis” (older adolescents only)	−-	−-	−-	−-	−-	0.852 (0.359)	0.853 (0.354)	0.867 (0.346)	0.822 (0.383)	0.833 (0.381)	0.884 (0.321)	0.667 (0.500)	0.741 (0.442)	0.857 (0.378)	0.860 (0.351)
**Total sample size**	996	143	853	89	512	54	341	72	419	71	434	21	134	7	43

Notes: *This table presents the overall means (and standard deviations) for responses on social connectedness and social cohesion outcomes for Hajati beneficiaries and non-beneficiaries collected during the Covid R2 phone survey.*

*Note that disability status is unknown for 4 adolescents and marital status is unknown for 1 adolescent girl.

± Note that the means presented exclude any adolescents who responded “don’t know” or “refused” for the relevant question. Note that the final outcome is restricted to the older cohort only (15–18 years), and the sample size is 395 (including 195 girls [30 beneficiaries], 196 boys [24 beneficiaries], 63 adolescents with disabilities [9 beneficiaries], and 50 adolescent girls who are currently or previously married [7 beneficiaries]).

Older cohort adolescents generally reported high levels of social cohesion in their communities; more than 8 in 10 agreed or partially agreed that people from diverse backgrounds in their community were “coming together to help each other during the current health crisis”.

Although sample sizes were too small for formal comparison, we also present mean responses among adolescents with disabilities, and adolescent girls who were currently (or had been) married, by *Hajati* status (Table 3). With the caveat that sample sizes are small, adolescents with disabilities in beneficiary households reported higher rates for several social connectedness and resilience outcomes compared to those in non-beneficiary households, including having a trusted friend (71 % vs 49 %), having a trusted adult (76 % vs 65 %), self-report of coping well with pandemic stress (81 % vs 54 %), and scoring in range for high resilient coping (33 % vs 15 %). Notably, married adolescent girls overall appeared to have lower peer connectedness than unmarried girls, and were less likely to have a trusted friend (43 % among *Hajati* beneficiaries; 47 % among non-beneficiaries compared to 56 % in the sample overall).

In formal comparisons, we find some evidence of improved outcomes for social connectedness and resilience associated with *Hajati* beneficiary status. First, we present the results of the multivariate regression overall and by subgroup, focusing on the effect size associated with household *Hajati* beneficiary status after controlling for other covariates (Table 4). Full regression results are provided in the appendix (Table S2 through S6).

**Table 4:** Multivariate regression results: effect size associated with *Hajati* beneficiary status.

**Outcome**±	All adolescents	Younger adolescents (age 12–14)	Older adolescents (age 15–18)	Adolescent girls	Adolescent boys
	coeff (se)	coeff (se)	coeff (se)	coeff (se)	coeff (se)
=1 if adolescent reports they have a friend that they can trust	−0.007	0.002	−0.020	−0.017	0.001
	(0.046)	(0.060)	(0.071)	(0.064)	(0.067)
=1 if adolescent reports they have an adult that they can trust	0.019	0.119*	−0.136	0.084	−0.038
	(0.045)	(0.056)	(0.074)	(0.062)	(0.066)
=1 if adolescent's friends are helping them cope with the pandemic	0.061	0.076	0.042	0.114	0.001
	(0.044)	(0.056)	(0.071)	(0.062)	(0.064)
=1 if other adults outside the family are helping them cope with the pandemic	0.036	0.005	0.085	0.145*	−0.092
	(0.047)	(0.060)	(0.074)	(0.065)	(0.064)
=1 if family is helping them to cope with pandemic stress	0.076*	0.047	0.128**	0.151***	0.004
	(0.030)	(0.041)	(0.041)	(0.036)	(0.047)
=1 if adolescent is coping well with pandemic stress	0.122**	0.140**	0.104	0.161**	0.083
	(0.041)	(0.052)	(0.067)	(0.055)	(0.061)
BRCS total score (0–16)	0.536	0.651	0.394	1.076**	0.036
	(0.274)	(0.341)	(0.464)	(0.384)	(0.389)
=1 if adolescent scored within low resilient coping range (0–9), BRCS	−0.076	−0.086	−0.073	−0.084	−0.077
	(0.041)	(0.057)	(0.059)	(0.059)	(0.058)
=1 if adolescent scored within high resilient coping range (13–16), BRCS	0.01	0.017	−0.003	0.087	−0.072
	(0.034)	(0.043)	(0.056)	(0.052)	(0.044)
=1 if adolescent agrees or partially agrees with the statement: “People in my community, regardless of nationality, ethnicity, and religion, are coming together to help each other during the current health crisis” (older adolescents only)	−-	−-	0.008	0.040	−0.023
	−-	−-	(0.053)	(0.068)	(0.086)
**Total sample size**	981	588	393	497	484

Notes: Each cell in this table presents the coefficient (with standard errors in parentheses) associated with *Hajati* beneficiary status drawn from a multivariate regression controlling for: gender, age cohort, disability status, marital status, baseline asset decile, baseline household size, and whether the household was female-headed at baseline. Complete regression results are presented in Supplementary Tables S2, S3, S4, S5 and S6. Statistically significant results are indicated as follows: * p < 0.05, ** p < 0.01, *** p < 0.001.

± Note that the models presented exclude any adolescents who responded “don’t know” or “refused” for the relevant question (outcome) or for the set of covariates used in the model. Note that the final outcome is restricted to the older cohort only (those aged 15–18 years), and the sample size is 393 (199 girls and 194 boys).

Overall, benefiting from *Hajati* appears to be associated with 2 of the 10 social connectedness and resilience outcomes, specifically those outcomes related to resilient coping and within-household connectedness. In our model that includes all adolescents, we find that *Hajati* beneficiary status is associated with an 8-percentage point increase in the likelihood of the adolescent reporting that their family is helping them to cope with the stress of the pandemic (p < 0.05). *Hajati* is associated with a larger increase for older adolescents (13 percentage points, p < 0.01) and adolescent girls (15 percentage points, p < 0.001), but no effect is seen when looking at younger adolescents only or adolescent boys only.

Furthermore, *Hajati* beneficiary status is associated with a 12-percentage point increase in the likelihood of the adolescent reporting that they are coping well with pandemic-related stress (p < 0.01). In our subgroup analyses, among younger adolescents only and adolescent girls only, *Hajati* is associated with slightly larger increases (14 and 16 percentage points respectively, p < 0.01 for both models). Additionally, adolescent girls from beneficiary households scored, on average, 1.076 points higher on the BRCS measure of resilience than peers from non-beneficiary households (a statistically significant increase at p < 0.01).

*Hajati* beneficiary status was associated with improved social connectedness outcomes outside of the household on one additional measure each among adolescent girls and younger adolescents. When the analysis is restricted to adolescent girls only, *Hajati* is associated with a 15-percentage point increase in girls reporting that “an adult outside of the household is helping to cope with the stress of the pandemic” (p < 0.05). Furthermore, when the analysis is restricted to younger adolescents only (aged 12–14), *Hajati* is associated with a 12-percentage point increase in the adolescent reporting that they have at least one adult who they can trust (although it should be noted that this person may be within or outside of the household) (p < 0.05).

On the other hand, we observed no differences by *Hajati* status on the adolescent’s likelihood of having trusted friends or on reporting that their friends are helping them to cope with the pandemic, indicating that *Hajati* may have limited impact on adolescent peer connections during a time of social distancing. Although adolescent girls who benefited from *Hajati* showed a small but significant increase in resilient coping compared with non-beneficiaries, resilience scores largely did not vary by beneficiary status. Finally, we did not see a significant difference in adolescent perception of social cohesion by *Hajati* beneficiary status.

In addition to multivariate regression, we also examined outcomes by *Hajati* beneficiary status after utilising the nearest-neighbour matching process (Table 5). These results are generally consistent with findings from the multivariate regression. First, adolescents in beneficiary households were 8 percentage points more likely to report that their family was helping them to cope with pandemic stress (p = 0.008) than matched peers in non-beneficiary households. This rate was higher among adolescent girls, where *Hajati* status was associated with a 17-percentage point increase (p < 0.001), as well as among older adolescents, where beneficiary status was associated with a 10–percentage point increase (p = 0.023).

**Table 5:** Average treatment effect (ATE) of *Hajati* on outcomes of interest at Covid R2 phone survey using nearest-neighbour matching, Syrian adolescents living in host communities.

**Outcome**±	All adolescents	Younger adolescents (age 12–14)	Older adolescents (age 15–18)	Adolescent girls	Adolescent boys
	coeff (se)	coeff (se)	coeff (se)	coeff (se)	coeff (se)
=1 if adolescent reports they have a friend that they can trust	−0.001 (0.053)	0.021 (0.072)	−0.038 (0.074)	−0.039 (0.079)	0.032 (0.073)
=1 if adolescent reports they have an adult that they can trust	−0.002 (0.052)	0.099 (0.065)	−0.14 (0.085)	0.044 (0.073)	−0.052 (0.073)
=1 if adolescent's friends are helping them cope with the pandemic	0.035 (0.054)	0.063 (0.069)	−0.016 (0.087)	0.083 (0.081)	−0.013 (0.072)
=1 if other adults outside the family are helping them cope with the pandemic	0.014 (0.053)	−0.002 (0.067)	0.034 (0.090)	0.138 (0.078)	−0.105 (0.073)
=1 if family is helping them to cope with pandemic stress	0.083 (0.031)**	0.070 (0.043)	0.105 (0.046)*	0.167 (0.035)***	0.001 (0.051)
=1 if adolescent is coping well with pandemic stress	0.135 (0.045)**	0.158 (0.057)**	0.106 (0.074)	0.159 (0.065)*	0.113 (0.061)
BRCS total score (0–16)	0.644 (0.328)	0.994 (0.382)**	0.044 (0.595)	1.353 (0.415)**	−0.046 (0.457)
=1 if adolescent scored within low resilient coping range (0–9), BRCS	−0.111 (0.048)*	−0.134 (0.064)*	−0.062 (0.071)	−0.146 (0.065)*	−0.076 (0.066)
=1 if adolescent scored within high resilient coping range (13–16), BRCS	0.030 (0.039)	0.072 (0.057)	−0.040 (0.047)	0.110 (0.067)	−0.049 (0.047)
=1 if adolescent agrees or partially agrees with the statement: “People in my community, regardless of nationality, ethnicity, and religion, are coming together to help each other during the current health crisis” (older adolescents only)	−-	−-	−0.024 (0.061)	−-	−-
**Total sample size**	981	588	393	484	497

Notes: Each cell is the coefficient (and standard error) from a separate regression and provides an estimate of the difference in the outcome between matched *Hajati* beneficiaries and non-beneficiaries. Estimates are derived from matching techniques that utilise the ‘teffects’ command in Stata, which computes robust Abadie-Imbens standard errors, to implement nearest-neighbour matching (using two matches per observation) to match beneficiaries to non-beneficiaries on a set of baseline household characteristics (asset decile, household size, and whether the household is female-headed) as well as adolescent characteristics (gender, age cohort, disability status, and adolescent marital status). We require exact matches for gender and age cohort. We repeat this matching analysis among younger adolescents only, older adolescents only, adolescent girls only, and adolescent boys only, matching on the same criteria (as relevant) to examine differences in outcomes associated with *Hajati* beneficiary status within subgroups.

± The sample sizes used for this analysis are slightly smaller than the total described in this analysis (n = 996) because any adolescents who responded “don’t know” or “refused” for a given question are excluded from the analysis for that outcome. Note that the final outcome is restricted to the older cohort only, and the sample size in the final analysis is 393 (199 girls and 194 boys). We do not present the disaggregated results by gender for the final outcome (which only includes the older cohort) because the smaller sample size does not lend itself to the nearest-neighbour matching method.

Second, beneficiary adolescents were nearly 14 percentage points more likely to report coping well with pandemic stress compared with non-beneficiary peers (p = 0.003), and this was particularly the case for younger adolescents and adolescent girls. Looking at the formal resilience outcomes, adolescents from beneficiary households were 11 percentage points less likely to score in the low resilience range on the BRCS compared with matched non-beneficiaries (p = 0.019). Among younger adolescents, *Hajati* beneficiary status was associated with a 0.9-point increase in resilience score on the 16-point BRCS (p = 0.009); younger adolescents in beneficiary households were also 13 percentage points less likely to have a low resilient coping score (p = 0.037). *Hajati* beneficiary status was associated with an even larger increase in resilience (1.4 points) among adolescent girls (p = 0.001).

However, in contrast to previous results, the matching analysis did not find significant differences in social connectedness outcomes related to relationships outside of the household across subgroups. Furthermore, we do not observe a significant difference among older adolescents in perceptions of social cohesion in the community during the pandemic; as previously noted, adolescents were highly likely to agree with the statement that people of diverse backgrounds in the community were supporting each other during the pandemic (85 % across beneficiaries and non-beneficiaries). Finally, in restricting our analysis to adolescent boys, we find no difference in any social connectedness or resilience outcomes associated with *Hajati* beneficiary status.

### 5.1 Insights from qualitative data

Turning to the qualitative findings, the in-depth interviews complement the survey data and provide insights into the pathways through which the *Hajati* cash transfer and the linked *Makani* empowerment programming are mediating adolescent resilience and psychosocial well-being. Although *Hajati* is a ‘labelled’ transfer,^2^ rather than being explicitly designed as a cash-plus initiative,^3^ many of the young people in the qualitative sample whose households were beneficiaries had also been involved at some point in UNICEF Jordan’s *Makani* programme. In particular, the qualitative findings demonstrate how cash-plus programming can facilitate stronger peer networks and improve social cohesion for refugee adolescents in host communities.

In line with the survey findings, the in-depth interviews indicated that the cash transfer is helping refugee adolescents to cope with the additional stressors of the pandemic by providing some relief to household economic stress. As a 15-year-old Syrian refugee girl explained, the cash enabled children to participate in online learning during lockdown:

*If we did not receive support from Hajati, my family would not be able to afford internet… The cash helps us to buy stationery and internet, and to keep learning…*

The qualitative findings also shed light on gender differences in terms of adult support for beneficiary adolescents’ coping abilities. Interviews suggested that adult support, especially from teachers and mentors, was relatively more important to girls as they had less access to peers during the pandemic (conservative gender norms that restrict girls’ mobility became even more stringent during lockdown). A 12-year-old Syrian refugee girl noted that she relied heavily on support from her teacher to deal with the stresses of online learning:

*I asked the teacher to explain the answers again for me… she explains it, audio records it, then sends it to me.*

Other girls reported valuing the emotional support they received from *Makani* centre mentors. One 16-year-old Syrian refugee girl explained that online communication between her friends and the *Makani* mentor helped to mitigate the social isolation of lockdown:

*I use WhatsApp, my friends and I created a group… and to communicate with the Makani centre facilitator… I feel less alone.*

Another 17-year-old Syrian refugee girl felt that her *Makani* mentor was more like a sister than a teacher, and provided valuable support:

*We cannot call her teacher… No, she is closer than sisters… At that time [during the pandemic], I needed someone to support me… moral support, a gift, telling me that you are a good person and you will pass all of that, and this is only temporary and you will pass it. Indeed, I wanted someone to help me to pass [get through] it…’R’ [Makani mentor] supported me very much and she told me that I can pass it normally with the least losses, and I actually did… I used to talk to her about things that happened and bothered me, we used to analyse them together*…

The qualitative findings also suggest that girls’ access to adolescent-focused programming before the pandemic influenced their connectedness and resilience during lockdowns, often due to closer pre-existing emotional ties to *Makani* mentors. A 12-year-old Syrian refugee girl emphasised that she was able to stay in close contact with her teacher after the lockdown started based on the trusting relationship the teacher had previously built up with her students:

*She [the facilitator] always advises us on what are the good things we should do. If someone was having trouble at home or someone was having an argument, we had to tell her, and she won’t tell anyone about it. She was our secret keeper. She would later talk to our parents and discuss that they are doing something that affects us so that our relationship becomes stronger. We can also talk to her about things that happen in school and she was able to fix them… I used to talk to her about how some girls used to beat me up, she used to go and make them apologise to me.*

Some girls also explained that the pandemic had brought relatively limited changes to their daily life, as girls were more used to being at home before the pandemic compared with their male peers. As a 13-year-old Syrian refugee girl noted:

*Prior to the corona[virus] I would come back from school, talk with my family a little, watch a TV series, help my mother… And now, with corona, it is the same… I spend my time with my family at home…*

This was in stark contrast to boys, who consistently emphasised the drastic changes in their lives since the onset of the pandemic. A 16-year-old Syrian refugee boy explained that his socio-emotional networks had been disrupted dramatically:

*It affected us so, so much… It kept me and my friends apart… I miss them so much, and I miss my school… I feel that I am alone in this life… I used to be extremely happy… My friends are like brothers to me… We used to go play football, entertain ourselves and laugh together… By the time of Covid-19 and curfew, we were no longer able to go to school nor see each other… This affected me and felt like a fish out of water – alone, having no one to get my back or entertain me.*

It is also important to note that although the survey findings indicated that *Hajati* beneficiaries were more likely than non-beneficiaries to report feeling emotionally supported by their parents during the pandemic, the qualitative interviews showed that parents could also be a source of stress for some adolescents due to the greater control they sought over their children. A 14-year-old Syrian refugee girl noted that her mobility was fully curtailed:

*My parents control me more now than before the pandemic… They prevented me from leaving the house…*

Several parents also admitted that the pandemic had led them to be overly protective of their children, not least because it exacerbated the vulnerability they already felt as refugees. As one Syrian mother of an adolescent explained:

*I do not allow my children to leave the house, I hide the keys… I allow them to leave only in the morning and I must be with them. I tell my children that if they leave the house, the police will take them. I want to protect them and protect others from [coronavirus] infection… all day long they quarrel with each other.*

A *Makani* mentor concurred, noting that parental restrictions were a major concern for adolescents during lockdown. Parents suddenly had much more frequent interactions with their children, and while some young people could retreat into an online world, for the most vulnerable individuals, this was often not possible:

*This age group is… the start of coping with peers. So, this lockdown caused big pressure on them as during this age they start to see the world and to be open to the world… But with corona[virus], the adolescents were forced to stay at home… The father and mother didn’t see the adolescent for a long time period previously… So, suddenly there is a process of observation that is too intense, especially on the boys. So, this affects them negatively because this restricts their movement… not all young people have Facebook, not all of the young people have internet bundles and not all of them can recharge their lines by the mobile cards to use Facebook to communicate…*

There was, however, an important difference between unmarried and married girls. Whereas interviews with unmarried girls pointed to the key role of adult support, married girls emphasised that they needed to draw on their own internal emotional reserves to support their husband, and that they were generally isolated from their family and could no longer rely on them for psychosocial support. A 17-year-old married Syrian refugee girl explained her situation as follows:

*Before corona[virus] issue, I used to go out and back, to visit my family and to visit my father-in-law’s family, while now I am staying at home in the quarantine. I haven’t seen my parents since we started the quarantine… My husband used to go to work in the beginning of the quarantine… A person who is adapted to work, he feels bored, and he wanted only to go out. So, this period was very difficult. When we were sitting together, he was very nervous… I can’t be nervous with him… [or it would add to] the problem. I don’t tell him: ‘I am sitting at home, same as you’, no. I allow him to talk to release his feelings… After, we understand each other. I tell him: ‘This is normal!*… *What shall we do? It… will pass, you will go back to your work, and you will be able to go out, if God wants’.*

Furthermore, the qualitative interviews underscored that peers played a key role in helping boys as well as girls to cope with the stresses of the pandemic in particular, and with forced displacement more generally. For some young people, peer support emerged organically and was not associated with participation in the *Makani* programme. Interacting with peers constituted a way to cope with the tight control and worries that parents had about their children’s well-being, which for refugee households was exacerbated by the precarity of their situation in Jordan. A 16-year-old Syrian refugee boy explained that he spent most of the time during lockdown away from his parents and at his cousins’ home because he craved contact with young people his own age, even though he recognised that his actions stressed his parents due to the risk of being caught by the police:

*My relationship with my parents deteriorated during corona[virus]… I am a nervous person so I get angry with them sometimes… They say to me ‘why do you go outside at night…?’ I can’t stay home, it drives me crazy staying home. I get so bored. So, I go out, stay up, spend time outside home… even during the curfew… and come back at sleeping time… I go to my aunt’s house and sit with her sons… We play on mobiles… But my family are afraid… They say ‘if the police caught you and take you to prison, we don’t have money to pay the 100 dinars fine to get you out*…*’.*

Younger adolescents and especially girls, who had less access to online networks, also highlighted how much they missed contact with peers, and that parental stress had a spillover effect on their own psychosocial well-being. A 12-year-old Syrian refugee girl explained:

*I can't go to school and go outside home and always there are diseases, also I don't see my friends… my family are not going outside a lot, and they stay frightened… my mother is always nervous, and… always angry with us… when would the school come back! because really, school is better.*

For some young people, *Makani* played an important role in linking them to peer networks that were then sustained through online and hybrid programming approaches during the pandemic. For those children and young people who had participated in *Makani* in the past or were still doing so, the one-stop community centres appeared to play a key role in facilitating those peer networks, especially for girls. A 15-year-old Syrian refugee girl explained that *Makani* had enabled her to make friends, including with Jordanians – relationships she had been able to maintain during the pandemic:

*I made new friends, during the [makani] programme I met two new friends… Yes, our friendship has lasted so far, I took their phone numbers to communicate with them… we were Syrian and Jordanian, there was no discrimination between us and we did not feel that there is a difference.*

Moreover, the qualitative findings also indicate the powerful role of *Makani* centres in fostering social cohesion among host and refugee communities, by bringing young people from both communities together and teaching about the importance of non-violence, as well as developing positive communication and negotiation skills. A 12-year-old Syrian refugee girl participating in *Makani* noted:

*I have friends of many nationalities… It’s a strong relationship. It’s not nice to discriminate against the person because of their nationality. Nationalities don’t differ, we are all the same… I liked to form friendships and learn more about them… I don’t know a lot about Jordan so being friends with Jordanians is helpful because they are able to tell me more about all the things I don’t know. I started to understand their dialect more as well… Iraqi, Jordanian… this made our friendship stronger…*

The value of such programming in promoting tolerant and inclusive attitudes should not be underestimated. Reflections from young people in our sample who had not participated in *Makani* underscored that discrimination against refugees and tensions in the host community can be very detrimental to young people’s psychosocial well-being. An 18-year-old Syrian refugee young woman emphasised that she fears speaking out and asserting her rights because of negative attitudes towards refugees among her classmates:

*We have 35 students, but we have only 3 Syrian students [in our class]. The remaining students are Jordanians. It is difficult for us to speak a word against them. They will destroy us… We feared to speak any word.*

A 15-year-old Syrian refugee girl further explained that not only do they encounter tensions among classmates but also that they are sceptical that they will receive support from school administrators due to exclusionary attitudes within the formal school system:

*There are many differences in the governmental school… As an example: she is Jordanian and I am a Syrian and we have a fight. So, she will have the right against me. They take her out of the problem and they consider that she has no guilt… They consider the guilty one is the Syrian…*

## 6 Discussion and conclusion

Overall, our findings align with the literature, which indicates that social protection programmes provided by the United Nations (UN) are critical to the basic well-being of the most vulnerable Syrian refugees in Jordan (Abu Hamad et al., 2017). The same source indicates that 99 % of respondents in UN post-distribution monitoring confirmed that cash assistance had improved their living conditions and reduced their financial burden. When cash assistance is sufficient (in terms of the amount) and complemented with other services, it helps to retain adolescents in school, and reduce child labour and child marriage (Abu Hamad et al., 2021, Abu Hamad et al., 2019).

Our findings indicate some modest but positive signals about the contribution of *Hajati* to adolescents’ social connectedness and resilience outcomes across subgroups. Multiple methods of analysis suggest that *Hajati* beneficiary households report higher levels of the horizontal dimensions of social connectedness, including small but significant increases in caregivers being able to help their children cope with pandemic-related stresses (90 % vs 84 %). *Hajati* beneficiary status was also associated with higher rates of adolescents reporting coping well with pandemic stress, and with improved measures of resilience among younger adolescents (aged 12–14) and adolescent girls. Our multivariate regression results also show that beneficiary status was associated with an increase in the rate at which adolescent girls reported that adults outside of the household were helping them to cope with pandemic-related stresses (15 percentage points). This was further reinforced by the qualitative findings, which emphasised the programme’s key role in supporting social connectedness with teachers and *Makani* mentors, who provided adolescents with psycho-emotional support. These findings in turn align with the evidence suggesting that cash transfers can have positive effects on adolescents’ broader well-being, including resilience and social connectedness (Jeong and Trako, 2022, Lowe et al., 2022, Zintl and Loewe, 2022).

One area in which the survey found no significant differences among *Hajati* beneficiaries and non-beneficiaries in terms of perceived levels of peer support during the pandemic. Yet our qualitative findings suggest that peer networks were critical to young people’s resilience more generally, and that linkages with the *Makani* empowerment programming were important in facilitating these peer interactions, especially for girls. Furthermore, the qualitative findings underscore that peer networks – especially those fostered by *Makani* programming – played a key role in helping boys as well as girls to cope with pandemic-related stresses and in promoting a sense of social cohesion among young people in host and refugee communities.

The extent to which social protection programmes can mitigate vulnerabilities and promote social cohesion is shaped by the design of each programme, its target group(s), monetary amount, and complementary services. In our study, the limited effects of social protection on vertical forms of social connectedness and social cohesion, as captured in the survey findings, could be attributed to the limited capacity of the cash programmes to meet refugees’ broader needs. The literature argues that cash transfers need to meet a certain income threshold to meaningfully support pathways out of poverty (de Groot et al., 2017, Tepperman, 2016). Our findings suggest that while the transfer amounts in Jordan can help support refugees’ survival needs (Abu Hamad et al., 2021), they appear to be too low to adequately address other challenges facing refugee households, especially those related to social connectedness with peers and social cohesion, and especially for older adolescents. The limited effect of cash transfers on social cohesion can also be attributed to a range of other factors, including refugees’ greater vulnerability, to differences in programme design and differences in entitlements, to the actors involved, the nationality of those who are eligible, and the availability of funding.

The qualitative findings, however, indicate that combining economic support through cash transfers with empowerment programming may deliver social cohesion gains specifically for adolescents in contexts of forced displacement. In line with the broader literature, which suggests that appropriately designed social protection programmes can foster participation and improve trust and inclusion among community members and between citizens and the state (Burchi et al., 2022, Mahmud and Sharpe, 2021), our qualitative interviews with adolescents suggested that *Makani* participation played an important role in fostering friendships across nationality divides, and in promoting inclusive attitudes.

## 7 Policy implications

Taken together, the findings indicate that social protection programming that takes a cash-plus approach – going beyond cash transfers to provide additional empowerment programming – may deliver better outcomes for adolescents in terms of supporting peer connectedness and social cohesion. Cash assistance alone cannot fully mitigate or address refugees’ complex vulnerabilities or ensure social cohesion with host communities. Our findings support the argument that assistance should not only focus on tackling economic vulnerability but also the social aspects of vulnerability. Social assistance programmes should therefore be conceptualised as part of a broader integrated social protection system and linked to complementary services designed to strengthen social cohesion (such as encouraging public dialogue and discussion, social solidarity, and fostering a sense of civic responsibility among beneficiaries and non-beneficiaries). It is therefore vital to deliver aid in a manner that supports both refugees and host communities, through national service providers wherever possible (European Commission, 2019).

As such, it is important to work with UN and national social protection partners to orient programme managers and social actors on how social protection programmes can foster social connectedness, community cohesion and peacebuilding processes, and to incorporate those goals within programme design and implementation. UN assistance, despite being vital to aid survival, cannot on its own mitigate or address the complex vulnerabilities facing populations affected by forced displacement. It is essential to integrate humanitarian and development programming to tackle both the economic and social aspects of vulnerability. This includes increasing employment opportunities, investing in education, and in complementary empowerment programmes for adolescents and young people, both to enhance individual resilience and promote social connectedness. Cash assistance should be part of a larger services network that includes referrals to specialist services for the most vulnerable individuals, and opportunities to build networks with peers and trusted adults. There is also a pressing need to invest in interventions to strengthen community cohesion such as organising awareness sessions, building communication and negotiation skills, and encouraging dialogue and networking among adolescents and caregivers. These should be approached as an integral part of programme design and effectiveness rather than as an optional extra.

It is vital to invest in cash-plus approaches that target both those directly affected by displacement and vulnerable households in the host communities in order to reduce community tensions, especially as conflicts become increasingly protracted (as is the case with the Syrian refugee crisis in the Middle East and North Africa region). Twinning cash transfers (which help to address economic vulnerabilities) with age-tailored empowerment programming for adolescents and young people (which addresses social vulnerabilities), and facilitating improved communication and peaceful co-existence between refugee and host communities, can deliver synergies. As such, it can also be part of broader efforts to develop transformative social protection programmes that promote social inclusion and sustainable peace, and to realise the promise of the SDGs, including SDG 16, which aims to promote just, peaceful and inclusive societies.

## 8 Data accessibility statement

The data collected in this study is subject for sharing according to the Gender and Adolescence: Global Evidence (GAGE) programme’s data-sharing policy. Potential users can contact the GAGE programme hub office to enquire about use ahead of public archiving. gage@odi.org.uk.

## 9 Consent to publish

All authors have read and approved the submission of this manuscript for publication.

## CRediT authorship contribution statement

**Bassam Abu Hamad:** Conceptualization, Writing – original draft. **Nicola Jones:** Conceptualization, Funding acquisition, Methodology, Supervision, Writing – original draft, Writing – review & editing. **Shoroq Abuhamad:** Data curation, Formal analysis. **Sarah Baird:** Conceptualization, Formal analysis, Funding acquisition, Writing – original draft, Writing – review & editing. **Erin Oakley:** Conceptualization, Formal analysis, Methodology, Validation, Writing – original draft.

## Funding

The World Health Organization’s Regional Office for the Eastern Mediterranean, the Ford Foundation, the Bill & Melinda Gates Foundation (# INV-003527) awarded through the National Bureau of Economic Research (NBER), and UK Aid from the UK Government provided funds to carry out this research.

## Declaration of competing interest

The authors declare that they have no known competing financial interests or personal relationships that could have appeared to influence the work reported in this paper.

## Data Availability

Data will be made available on request.

## Footnotes

1 We include household assets as adolescents are typically embedded within households and we want to factor in household economic status as one variable that could shape adolescent experiences of social connectedness.

2 ‘A labelled cash transfer communicates to beneficiaries the intended purpose of the cash transfer but does not threaten expulsion from the programme if that behaviour is not fulfilled (see, for example, Riley, 2020).

3 ‘Cash-plus’ refers to a diverse set of programmes, most commonly the linking of social assistance (specifically cash transfers) with information, services, training, social networks or social norms activities, and other support aimed at tackling multidimensional social and economic risks and vulnerabilities (Roelen et al., 2017).
