# Supplementary material for: Can social protection contribute to social connectedness in contexts of forced displacement and crisis? Lessons from Jordan’s labelled cash transfer for education
Source: World Dev. 2025 Apr;188:106886. doi: 10.1016/j.worlddev.2024.106886 (PMC11803516; doi:10.1016/j.worlddev.2024.106886)
Supplement: Supplementary Data 1 [file mmc1.docx]

**Appendix**

**Table S1. Attrition Analysis**

| **Adolescent/household characteristics** | **Coefficient (95% confidence interval)** | **p-value** |
| --- | --- | --- |
| Female adolescent | -0.038 (-0.085, 0.010) | 0.122 |
| Younger cohort (age 10-14 at baseline) | -0.057 (-0.109, -0.006) | 0.029 |
| Adolescent with disabilities (baseline status) | 0.020 (-0.038, 0.078) | 0.502 |
| Adolescent was ever married at baseline | -0.012 (-0.156, 0.133) | 0.876 |
| Household had above-median assets at baseline | 0.021 (-0.028, 0.071) | 0.394 |
| Female head of household | -0.027 (-0.083, 0.029) | 0.347 |
| Adolescent was enrolled in school at baseline | 0.034 (-0.040, 0.108) | 0.372 |

Notes: This table presents an attrition analysis for adolescents targeted for the analysis presented in this manuscript. At baseline, we interviewed 1,319 eligible adolescents (Syrian refugees living in Host Communities in Jordan and who would be the appropriate age at the time of the Covid-19 Phone Survey). From this sample, we ultimately reached 996 adolescent-caregiver dyads. The model above presents the association between baseline adolescent and household characteristics on the likelihood of follow-up during the phone survey drawn from a linear probability model.

**Table S2. Multivariate regression results, all adolescents**

| **Covariates** | =1 if adolescent reports they have a friend that they can trust | =1 if adolescent reports they have an adult that they can trust | =1 if adolescent's friends are helping them cope with the pandemic | =1 if other adults outside the family are helping them cope with the pandemic | =1 if family is helping them to cope with pandemic stress | =1 if adolescent is coping well with pandemic stress | BRCS total score (0–16) | =1 if adolescent scored within low resilient coping range (0–9), BRCS | =1 if adolescent scored within high resilient coping range (13–16), BRCS | =1 if adolescent agrees with social cohesion statement (older adolescents only) |
| --- | --- | --- | --- | --- | --- | --- | --- | --- | --- | --- |
| =1 if Hajati beneficiary | -0.007 | 0.019 | 0.061 | 0.036 | 0.076* | 0.122** | 0.536 | -0.076 | 0.01 | **--** |
|  | (0.046) | (0.045) | (0.044) | (0.047) | (0.030) | (0.041) | (0.274) | (0.041) | (0.034) |  |
| =1 if adolescent is female | -0.009 | -0.003 | 0.008 | -0.061 | -0.034 | 0.022 | -0.385* | 0.022 | -0.029 | **--** |
|  | (0.032) | (0.031) | (0.032) | (0.032) | (0.024) | (0.031) | (0.191) | (0.031) | (0.023) |  |
| =1 if younger cohort (age 12-14) | -0.160*** | -0.106*** | -0.02 | 0.002 | 0.008 | -0.028 | -0.931*** | 0.137*** | -0.021 | **--** |
|  | (0.033) | (0.032) | (0.033) | (0.034) | (0.025) | (0.033) | (0.196) | (0.032) | (0.025) |  |
| =1 if adolescent has a disability | -0.058 | 0.014 | -0.076 | -0.054 | -0.021 | -0.093* | -0.318 | 0.063 | 0.018 | **--** |
|  | (0.043) | (0.042) | (0.044) | (0.043) | (0.033) | (0.043) | (0.274) | (0.042) | (0.033) |  |
| =1 if adolescent is ever married | -0.174* | 0.00 | -0.039 | 0.047 | 0.068 | 0.03 | 0.169 | -0.042 | 0.088 | **--** |
|  | (0.076) | (0.069) | (0.074) | (0.077) | (0.050) | (0.070) | (0.498) | (0.066) | (0.063) |  |
| Household baseline asset decile (1-10) | 0.008 | 0.006 | 0.011 | 0.001 | 0.007 | 0.003 | 0.076* | -0.009 | 0.005 | **--** |
|  | (0.006) | (0.006) | (0.006) | (0.006) | (0.004) | (0.006) | (0.035) | (0.006) | (0.005) |  |
| Household size | -0.008 | -0.003 | -0.009 | 0.002 | -0.007 | -0.002 | -0.028 | 0 | -0.006 | **--** |
|  | (0.006) | (0.006) | (0.006) | (0.006) | (0.005) | (0.006) | (0.034) | (0.006) | (0.004) |  |
| =1 if female-headed household | 0.035 | -0.081* | -0.016 | 0.007 | -0.027 | -0.014 | 0.021 | -0.017 | 0.009 | **--** |
|  | (0.038) | (0.038) | (0.038) | (0.039) | (0.029) | (0.037) | (0.227) | (0.036) | (0.029) |  |
|  | | | | | | | | | | |
| Constant | 0.696*** | 0.726*** | 0.659*** | 0.435*** | 0.875*** | 0.664*** | 10.735*** | 0.294*** | 0.192*** | **--** |
|  | (0.065) | (0.063) | (0.065) | (0.066) | (0.050) | (0.063) | (0.360) | (0.060) | (0.042) |  |
|  | | | | | | | | | | |
| Sample size^±^ | 981 | 981 | 981 | 980 | 980 | 980 | 980 | 980 | 980 | **--** |

Notes: Each cell in this table presents the coefficient (with standard errors in parentheses) for each covariate included in the multivariate regression: *Hajati* beneficiary status, gender, age cohort, disability status, marital status, baseline asset decile, baseline household size, and whether the household was female-headed at baseline. Statistically significant results are indicated as follows: * p<0.05, ** p<0.01, *** p<0.001.

^±^ Note that the models presented exclude any adolescents who responded ‘don’t know’ or ‘refused’ for the relevant question (outcome) or for the set of covariates used in the model. Note that the final outcome is restricted to the older cohort only (and thus not presented in Table S1).

**Table S3. Multivariate regression results, younger adolescents (age 12-14)**

| **Covariates** | =1 if adolescent reports they have a friend that they can trust | =1 if adolescent reports they have an adult that they can trust | =1 if adolescent's friends are helping them cope with the pandemic | =1 if other adults outside the family are helping them cope with the pandemic | =1 if family is helping them to cope with pandemic stress | =1 if adolescent is coping well with pandemic stress | BRCS total score (0–16) | =1 if adolescent scored within low resilient coping range (0–9), BRCS | =1 if adolescent scored within high resilient coping range (13–16), BRCS | =1 if adolescent agrees with social cohesion statement (older adolescents only) |
| --- | --- | --- | --- | --- | --- | --- | --- | --- | --- | --- |
| =1 if Hajati beneficiary | 0.002 | 0.119* | 0.076 | 0.005 | 0.047 | 0.140** | 0.651 | -0.086 | 0.017 | **--** |
|  | (0.060) | (0.056) | (0.056) | (0.060) | (0.041) | (0.052) | (0.341) | (0.057) | (0.043) |  |
| =1 if adolescent is female | 0.034 | 0.013 | 0.011 | -0.057 | -0.019 | 0.072 | -0.123 | -0.035 | -0.045 | **--** |
|  | (0.041) | (0.040) | (0.040) | (0.041) | (0.030) | (0.039) | (0.249) | (0.040) | (0.029) |  |
| =1 if adolescent has a disability | -0.063 | 0.080 | -0.050 | -0.004 | 0.008 | -0.096 | -0.522 | 0.095 | 0.043 | **--** |
|  | (0.056) | (0.054) | (0.056) | (0.057) | (0.041) | (0.056) | (0.391) | (0.057) | (0.044) |  |
| =1 if adolescent is ever married | 0.000 | 0.000 | 0.000 | 0.000 | 0.000 | 0.000 | 0.000 | 0.000 | 0.000 | **--** |
|  | (.) | (.) | (.) | (.) | (.) | (.) | (.) | (.) | (.) |  |
| Household baseline asset decile (1-10) | 0.014 | 0.004 | 0.005 | -0.004 | 0.006 | -0.001 | 0.043 | -0.005 | 0.001 | **--** |
|  | (0.008) | (0.008) | (0.008) | (0.008) | (0.006) | (0.008) | (0.047) | (0.008) | (0.006) |  |
| Household size | 0.004 | -0.006 | -0.021* | -0.001 | -0.018* | -0.015 | -0.098 | 0.015 | -0.006 | **--** |
|  | (0.009) | (0.009) | (0.009) | (0.009) | (0.008) | (0.009) | (0.054) | (0.009) | (0.005) |  |
| =1 if female-headed household | 0.072 | -0.053 | -0.038 | 0.005 | -0.005 | 0.000 | 0.011 | 0.021 | 0.012 | **--** |
|  | (0.053) | (0.052) | (0.052) | (0.053) | (0.037) | (0.050) | (0.317) | (0.052) | (0.038) |  |
|  | | | | | | | | | | |
| Constant | 0.395*** | 0.611*** | 0.745*** | 0.479*** | 0.950*** | 0.711*** | 10.342*** | 0.321*** | 0.190*** | **--** |
|  | (0.083) | (0.081) | (0.081) | (0.084) | (0.072) | (0.083) | (0.483) | (0.085) | (0.052) |  |
|  | | | | | | | | | | |
| Sample size^±^ | 588 | 588 | 588 | 587 | 588 | 587 | 587 | 587 | 587 | **--** |

Notes: Each cell in this table presents the coefficient (with standard errors in parentheses) for each covariate included in the multivariate regression: *Hajati* beneficiary status, gender, disability status, marital status, baseline asset decile, baseline household size, and whether the household was female-headed at baseline. Statistically significant results are indicated as follows: * p<0.05, ** p<0.01, *** p<0.001.

^±^ Note that the models presented exclude any adolescents who responded ‘don’t know’ or ‘refused’ for the relevant question (outcome) or for the set of covariates used in the model. Note that the final outcome is restricted to the older cohort only (and thus not presented in Table S2).

**Table S4. Multivariate regression results, older adolescents (age 15-18)**

| **Covariates** | =1 if adolescent reports they have a friend that they can trust | =1 if adolescent reports they have an adult that they can trust | =1 if adolescent's friends are helping them cope with the pandemic | =1 if other adults outside the family are helping them cope with the pandemic | =1 if family is helping them to cope with pandemic stress | =1 if adolescent is coping well with pandemic stress | BRCS total score (0–16) | =1 if adolescent scored within low resilient coping range (0–9), BRCS | =1 if adolescent scored within high resilient coping range (13–16), BRCS | =1 if adolescent agrees with social cohesion statement (older adolescents only) |
| --- | --- | --- | --- | --- | --- | --- | --- | --- | --- | --- |
| =1 if Hajati beneficiary | -0.020 | -0.136 | 0.042 | 0.085 | 0.128** | 0.104 | 0.394 | -0.073 | -0.003 | 0.008 |
|  | (0.071) | (0.074) | (0.071) | (0.074) | (0.041) | (0.067) | (0.464) | (0.059) | (0.056) | (0.053) |
| =1 if adolescent is female | -0.085 | -0.019 | 0.009 | -0.062 | -0.062 | -0.062 | -0.836** | 0.119* | 0.003 | -0.053 |
|  | (0.052) | (0.049) | (0.052) | (0.053) | (0.040) | (0.051) | (0.295) | (0.047) | (0.040) | (0.039) |
| =1 if adolescent has a disability | -0.038 | -0.076 | -0.118 | -0.129* | -0.059 | -0.079 | 0.007 | 0.010 | -0.023 | -0.145* |
|  | (0.067) | (0.066) | (0.070) | (0.066) | (0.055) | (0.067) | (0.361) | (0.061) | (0.050) | (0.060) |
| =1 if adolescent is ever married | -0.135 | 0.002 | -0.042 | 0.048 | 0.072 | 0.066 | 0.383 | -0.091 | 0.072 | 0.039 |
|  | (0.078) | (0.073) | (0.078) | (0.081) | (0.053) | (0.073) | (0.515) | (0.068) | (0.065) | (0.057) |
| Household baseline asset decile (1-10) | -0.002 | 0.009 | 0.019* | 0.009 | 0.006 | 0.006 | 0.112* | -0.014 | 0.010 | 0.002 |
|  | (0.009) | (0.009) | (0.010) | (0.010) | (0.007) | (0.009) | (0.050) | (0.008) | (0.008) | (0.006) |
| Household size | -0.019* | -0.001 | 0.002 | 0.005 | 0.004 | 0.011 | 0.043 | -0.015* | -0.006 | -0.003 |
|  | (0.008) | (0.009) | (0.008) | (0.009) | (0.005) | (0.008) | (0.042) | (0.007) | (0.005) | (0.006) |
| =1 if female-headed household | -0.012 | -0.112* | 0.012 | 0.017 | -0.060 | -0.037 | 0.020 | -0.057 | 0.011 | -0.019 |
|  | (0.055) | (0.055) | (0.056) | (0.058) | (0.046) | (0.056) | (0.323) | (0.049) | (0.045) | (0.042) |
|  | | | | | | | | | | |
| Constant | 0.866*** | 0.744*** | 0.540*** | 0.381*** | 0.821*** | 0.604*** | 10.223*** | 0.400*** | 0.161** | 0.910*** |
|  | (0.081) | (0.087) | (0.087) | (0.089) | (0.057) | (0.084) | (0.466) | (0.074) | (0.060) | (0.055) |
|  | | | | | | | | | | |
| Sample size^±^ | 393 | 393 | 393 | 393 | 392 | 393 | 393 | 393 | 393 | 393 |

Notes: Each cell in this table presents the coefficient (with standard errors in parentheses) for each covariate included in the multivariate regression: *Hajati* beneficiary status, gender, disability status, marital status, baseline asset decile, baseline household size, and whether the household was female-headed at baseline. Statistically significant results are indicated as follows: * p<0.05, ** p<0.01, *** p<0.001.

^±^ Note that the models presented exclude any adolescents who responded ‘don’t know’ or ‘refused’ for the relevant question (outcome) or for the set of covariates used in the model. Note that the final outcome is restricted to the older cohort only (those aged 15–18 years), and the sample size is 393 (199 girls and 194 boys).

**Table S5. Multivariate regression results, adolescent girls**

| **Covariates** | =1 if adolescent reports they have a friend that they can trust | =1 if adolescent reports they have an adult that they can trust | =1 if adolescent's friends are helping them cope with the pandemic | =1 if other adults outside the family are helping them cope with the pandemic | =1 if family is helping them to cope with pandemic stress | =1 if adolescent is coping well with pandemic stress | BRCS total score (0–16) | =1 if adolescent scored within low resilient coping range (0–9), BRCS | =1 if adolescent scored within high resilient coping range (13–16), BRCS | =1 if adolescent agrees with social cohesion statement (older adolescents only) |
| --- | --- | --- | --- | --- | --- | --- | --- | --- | --- | --- |
| =1 if Hajati beneficiary | -0.017 | 0.084 | 0.114 | 0.145* | 0.151*** | 0.161** | 1.076** | -0.084 | 0.087 | 0.040 |
|  | (0.064) | (0.062) | (0.062) | (0.065) | (0.036) | (0.055) | (0.384) | (0.059) | (0.052) | (0.068) |
| =1 if younger cohort (age 12-14) | -0.095 | -0.092 | -0.015 | 0.012 | 0.027 | 0.043 | -0.57 | 0.063 | -0.043 | **--** |
|  | (0.050) | (0.047) | (0.049) | (0.049) | (0.039) | (0.048) | (0.300) | (0.048) | (0.036) |  |
| =1 if adolescent has a disability | 0.035 | 0.015 | -0.055 | -0.002 | -0.011 | -0.038 | -0.135 | 0.048 | 0.061 | -0.109 |
|  | (0.061) | (0.059) | (0.063) | (0.062) | (0.048) | (0.060) | (0.410) | (0.061) | (0.049) | (0.083) |
| =1 if adolescent is ever married | -0.15 | -0.014 | -0.011 | 0.081 | 0.074 | 0.063 | 0.361 | -0.072 | 0.084 | 0.037 |
|  | (0.081) | (0.075) | (0.079) | (0.081) | (0.056) | (0.077) | (0.540) | (0.072) | (0.068) | (0.059) |
| Household baseline asset decile (1-10) | 0.013 | -0.001 | 0.008 | -0.006 | 0.01 | 0 | 0.056 | 0.003 | 0.005 | -0.002 |
|  | (0.009) | (0.008) | (0.008) | (0.009) | (0.007) | (0.008) | (0.051) | (0.008) | (0.006) | (0.011) |
| Household size | -0.012 | 0.008 | -0.012 | -0.005 | -0.006 | -0.001 | 0.013 | -0.008 | -0.006 | 0.005 |
|  | (0.010) | (0.009) | (0.010) | (0.010) | (0.008) | (0.010) | (0.049) | (0.009) | (0.005) | (0.010) |
| =1 if female-headed household | 0.041 | -0.118* | -0.067 | -0.009 | -0.06 | -0.01 | -0.355 | 0.041 | -0.026 | 0.024 |
|  | (0.055) | (0.054) | (0.054) | (0.055) | (0.044) | (0.053) | (0.335) | (0.054) | (0.038) | (0.062) |
|  | | | | | | | | | | |
| Constant | 0.641*** | 0.665*** | 0.693*** | 0.425*** | 0.799*** | 0.626*** | 9.886*** | 0.358*** | 0.163** | 0.797*** |
|  | (0.096) | (0.087) | (0.097) | (0.095) | (0.080) | (0.095) | (0.532) | (0.093) | (0.059) | (0.096) |
|  | | | | | | | | | | |
| Sample size^±^ | 484 | 484 | 484 | 483 | 484 | 484 | 483 | 483 | 483 | 199 |

Notes: Each cell in this table presents the coefficient (with standard errors in parentheses) for each covariate included in the multivariate regression: *Hajati* beneficiary status, age cohort, disability status, marital status, baseline asset decile, baseline household size, and whether the household was female-headed at baseline. Statistically significant results are indicated as follows: * p<0.05, ** p<0.01, *** p<0.001.

^±^ Note that the models presented exclude any adolescents who responded ‘don’t know’ or ‘refused’ for the relevant question (outcome) or for the set of covariates used in the model. Note that the final outcome is restricted to the older cohort only (those aged 15–18 years), and the sample size is 393 (199 girls and 194 boys).

**Table S6. Multivariate regression results, adolescent boys**

| **Covariates** | =1 if adolescent reports they have a friend that they can trust | =1 if adolescent reports they have an adult that they can trust | =1 if adolescent's friends are helping them cope with the pandemic | =1 if other adults outside the family are helping them cope with the pandemic | =1 if family is helping them to cope with pandemic stress | =1 if adolescent is coping well with pandemic stress | BRCS total score (0–16) | =1 if adolescent scored within low resilient coping range (0–9), BRCS | =1 if adolescent scored within high resilient coping range (13–16), BRCS | =1 if adolescent agrees with social cohesion statement (older adolescents only) |
| --- | --- | --- | --- | --- | --- | --- | --- | --- | --- | --- |
| =1 if Hajati beneficiary | 0.001 | -0.038 | 0.001 | -0.092 | 0.004 | 0.083 | 0.036 | -0.077 | -0.072 | -0.023 |
|  | (0.067) | (0.066) | (0.064) | (0.064) | (0.047) | (0.061) | (0.389) | (0.058) | (0.044) | (0.086) |
| =1 if younger cohort (age 12-14) | -0.213*** | -0.112* | -0.02 | 0.003 | -0.006 | -0.083 | -1.192*** | 0.191*** | 0.004 |  |
|  | (0.044) | (0.043) | (0.045) | (0.046) | (0.032) | (0.044) | (0.258) | (0.041) | (0.034) |  |
| =1 if adolescent has a disability | -0.140* | 0.013 | -0.101 | -0.116 | -0.032 | -0.143* | -0.488 | 0.07 | -0.03 | -0.191* |
|  | (0.061) | (0.060) | (0.062) | (0.060) | (0.046) | (0.061) | (0.368) | (0.057) | (0.045) | (0.087) |
| =1 if adolescent is ever married | 0.278*** | 0.327*** | -0.628*** | -0.462*** | 0.144*** | 0.318*** | 0.038 | -0.257*** | -0.175*** | 0.125** |
|  | (0.047) | (0.044) | (0.052) | (0.048) | (0.033) | (0.046) | (0.962) | (0.058) | (0.042) | (0.039) |
| Household baseline asset decile (1-10) | 0.003 | 0.014 | 0.013 | 0.008 | 0.005 | 0.006 | 0.103* | -0.022** | 0.006 | 0.007 |
|  | (0.008) | (0.008) | (0.008) | (0.009) | (0.006) | (0.008) | (0.048) | (0.008) | (0.007) | (0.007) |
| Household size | -0.004 | -0.01 | -0.005 | 0.01 | -0.007 | -0.002 | -0.044 | 0.005 | -0.004 | -0.009 |
|  | (0.009) | (0.008) | (0.008) | (0.008) | (0.007) | (0.008) | (0.048) | (0.008) | (0.005) | (0.008) |
| =1 if female-headed household | 0.023 | -0.05 | 0.039 | 0.029 | 0 | -0.022 | 0.366 | -0.076 | 0.042 | -0.069 |
|  | (0.053) | (0.053) | (0.053) | (0.055) | (0.038) | (0.053) | (0.300) | (0.047) | (0.042) | (0.058) |
|  | | | | | | | | | | |
| Constant | 0.734*** | 0.741*** | 0.623*** | 0.372*** | 0.892*** | 0.699*** | 10.912*** | 0.294*** | 0.175** | 0.958*** |
|  | (0.089) | (0.085) | (0.085) | (0.086) | (0.064) | (0.084) | (0.491) | (0.078) | (0.059) | (0.070) |
|  | | | | | | | | | | |
| Sample size^±^ | 497 | 497 | 497 | 497 | 496 | 496 | 497 | 497 | 497 | 194 |

Notes: Each cell in this table presents the coefficient (with standard errors in parentheses) for each covariate included in the multivariate regression: *Hajati* beneficiary status, age cohort, disability status, marital status, baseline asset decile, baseline household size, and whether the household was female-headed at baseline. Statistically significant results are indicated as follows: * p<0.05, ** p<0.01, *** p<0.001.

^±^ Note that the models presented exclude any adolescents who responded ‘don’t know’ or ‘refused’ for the relevant question (outcome) or for the set of covariates used in the model. Note that the final outcome is restricted to the older cohort only (those aged 15–18 years), and the sample size is 393 (199 girls and 194 boys).
